# Editorial: Immunological consequences of antigen sampling at mucosal surfaces, volume II

**DOI:** 10.3389/fimmu.2024.1499174

**Published:** 2024-10-11

**Authors:** Neil A. Mabbott, Koji Hase

**Affiliations:** ^1^ The Roslin Institute & Royal (Dick) School of Veterinary Studies, University of Edinburgh, Midlothian, United Kingdom; ^2^ Division of Biochemistry, Faculty of Pharmacy and Graduate School of Pharmaceutical Science, Keio University, Tokyo, Japan; ^3^ Institute of Fermentation Sciences (IFeS), Faculty of Food and Agricultural Sciences, Fukushima University, Fukushima, Japan; ^4^ International Vaccine Design Center, The Institute of Medical Science, The University of Tokyo (IMSUT), Tokyo, Japan

**Keywords:** intestinal epithelial cells, M cells, gastrointestinal tract, mucosal immunity, Peyer’s patches

Mucosal surfaces such as those in the gastrointestinal and respiratory tracts are continuously exposed to commensal and pathogenic microorganisms. The epithelial layers lining these mucosal surfaces help provide an important first line of defense against certain pathogens such as bacteria, viruses, parasites and their toxins. Specialized cells within the mucosal epithelia play important roles in the initial sampling and transportation of antigens across these surfaces to enable the mucosal immune system to provide host defense by generating specific immunity against potential pathogenic agents. At the same time, the mucosal immune system must also suppress immune responses, or display tolerance to commensal microbes and food components. The six papers that were published in the second volume of this Research Topic provide a useful update on the progress that has been made in our understanding of the diverse cellular mechanisms that mediate antigen sampling across mucosal epithelia. Some of these studies also highlight how these processes may be affected by certain infections or aging.

M cells are a unique population of mucosal epithelial cells that can transport particulate antigens acquired at their apical surface directly through to the specialized pocket beneath their basolateral surface: a process known as transcytosis. While M cells were originally identified in the intestinal tract (1), cells with M cell-like characteristics have since been described in the mucosal surfaces of many other tissues including the airways (2) and eye region (see Oya et al. below). Del Castillo and Lo provided an updated account of the characteristics of M cells, along with a useful description of other cell types in the intestine, oral cavity and thymus that share M cell-like features. Given this range of diversity the authors argued that M cells, like many other immune cells, are not a homogeneous population. Rather, they suggested that M cells represent a functional niche that can exist in different environments, which in turn influences their role.

Oya et al. extend the sites where cells with M cell-like characteristics have been described to include the ocular mucosal surface overlying the tear-associated lymphoid tissue (TALT) attached to the nasolacrimal sac. These cells share characteristic features with intestinal M cells, including the expression of uptake receptors for bacterial pathogens. Similar to M cells in Peyer’s patches, the differentiation of TALT M cells is regulated by RANKL-RANK signaling. Importantly, data from immunization experiments suggested that antigen uptake by TALT-associated M cells may play a role in immunosurveillance and the induction of specific mucosal immune responses in the eye region.

Gastrointestinal tumors are a major health issue. Sasaki et al. used a transgenic mouse model that spontaneously develops intestinal tumors to investigate the conditions that lead to their formation. They showed that the presence of food antigens in the gut plays an important role in suppressing tumorigenesis in the small intestine. When the mice were fed an antigen-free diet the incidence of these tumors was increased in their small intestine. In this system, M cells were shown to transcytose food-derived antigens into the small intestinal Peyer’s patches. Furthermore, in the absence of Peyer’s patches, the abundance of tumors was increased, and this coincided with a reduced abundance of T cells (Th1 cells, Treg cells and CD8^+^ T cells) in the small intestine lamina propria. Exactly how food antigens suppress small intestinal tumorigenesis remains to be determined, but the identification of food components that mediate this activity may provide a novel means of reducing disease incidence. Because the antigen-free diet used in this study shares similarities with clinically used enteral nutrition (3), questions remain concerning whether enteral nutrition may have a similar impact on gastrointestinal tumors in humans.

Each of the above studies provides examples of how M cells play an important role in the initial uptake and delivery of antigens across epithelial surfaces to induce mucosal immunity. However, antigen sampling is not exclusively restricted to these cells. For example, intestinal epithelial cells (IEC) in the epithelia overlying Peyer’s patches can acquire particles from the gut lumen (4), and goblet cell-associated passages can deliver low molecular weight soluble antigens to underlying conventional dendritic cells in the lamina propria (5). In their study, Cohen-Kedar et al. described how human IEC can sense and acquire the commensal fungal microorganism *Candida albicans*. Mechanistically they show that the phagocytosis of zymosan and *C. albicans* required interactions between the receptor Dectin-1 and fungal β-glucan, but occurred independently of Syk (spleen tyrosine kinase). This interaction was subsequently shown to lead to LC3-associated phagocytosis (LAP) and the degradation of *C. albicans* within lysosomes. These observations provide an important advance in our understanding of the role of IEC in immunosurveillance in the intestine.

Aging has a profound negative impact on the function of the mucosal immune system. This functional decline, known as immunosenescence, is accompanied by increased susceptibility of the elderly to gastrointestinal and respiratory infections. For example, the elderly are more susceptible to infection with the SARS-Cov-2 coronavirus with patients over 80 years of age having the highest incidence of severe COVID-19 disease and mortality (6). Aging also adversely affects the functional maturation of M cells in the small intestine. Aged mice have significantly fewer M cells in the epithelium overlying their Peyer’s patches (7). Donaldson et al. show that aging also affects the function of Paneth cells within the intestinal crypts. Since age-related disturbances to Paneth cells indirectly impede M cell differentiation (8), treatments that restore Paneth cell function may help improve M cell differentiation and mucosal immunity in the elderly.

Impaired epithelial cell regeneration is also associated with HIV infection, but little is understood about the cellular mechanisms that contribute to this. Boby et al. analyzed the intestines of simian immunodeficiency virus (SIV)-infected Rhesus macaques. Their data revealed that SIV infection is associated with disruptions in intestinal stem cell homeostasis and metabolism that negatively affect the regenerative capacity of the gut epithelium. Further mechanistic insights may aid in the development of novel therapeutics for the treatment of HIV-mediated enteropathy.

The Research Topic published in this second volume highlights some exciting advances in our understanding of antigen sampling at mucosal surfaces throughout the mammalian body. Further investigation and collaboration in this field may help to identify novel means to enhance the efficacy of mucosal vaccination, protect against mucosally-acquired infections and gastrointestinal tumors or counteract the adverse effects of aging on the mucosal immunity.
